# Comparison of the long-term outcome of home vs. inpatient treatment: 18–24 months follow-up of a non-randomized controlled trial

**DOI:** 10.1007/s00787-025-02677-x

**Published:** 2025-03-10

**Authors:** Daniel Graf, Stefan Lerch, Ulrich Böhnke, Corinna Reichl, Michael Kaess

**Affiliations:** 1 University Hospital of Child and Adolescent Psychiatry and Psychotherapy, University of Bern, Bolligenstrasse 111, Stöckli, Bern, 3000, 60 Switzerland; 2 Department of Child and Adolescent Psychiatry, Center for Psychosocial Medicine, University of Heidelberg, Heidelberg, Germany

**Keywords:** Home treatment, Therapy setting, Child and adolescent psychiatry, Psychotherapy research

## Abstract

Home Treatment (HT) in child and adolescent psychiatry is an increasingly important topic in the current context of global crises and strained mental health systems. We implemented a HT program provided by a multiprofessional treatment team and compared long-term outcomes of 27 patients (48% female, Ø15.15 ± 2.77 years) with those of 48 patients (69% female, Ø16.35 ± 2.87 years) who received inpatient treatment as usual (I-TAU). Psychopathology was assessed using the Health of the Nation Outcome Scale for Children and Adolescents (HoNOSCA[-SR]) and psychosocial functioning was assessed using the Global Assessment of Functioning (GAF) at admission, discharge, and 18–24 months after discharge. Treatment outcomes were analyzed using mixed models. The results showed that patients in the HT arm had significantly lower HoNOSCA scores at follow-up (*β* = -4.25 [95%CI: -7.64 to -0.86], *SE* = 1.73, *p* = 0.014) and higher GAF scores (*β* = 12.09 [95%CI: 4.48 to 19.70], *SE* = 3.88, *p* = 0.002) compared to those in the I-TAU arm. No significant differences were observed in HoNOSCA-SR scores (*β* = -2.46 [95%CI: -9.16 to 4.30], *SE* = 3.43, *p* = 0.48) and readmission rates (OR = 1.23 [95%CI = 0.47 to 3.20], *p* = 0.67). These results highlight the potential of HT in improving long-term functional and psychopathological outcomes in youth mental health. HT may be an equally effective and even more sustainable type of treatment for child and adolescent mental disorders. The trial was preregistered at the German Clinical Trials Register (DRKS00025424, 05/27/2021).

**Supplementary Information:**

The online version contains supplementary material available at 10.1007/s00787-025-02677-x.

## Introduction

Most mental disorders have their onset in childhood or adolescence [1, 2], with global point prevalence estimates approaching 14% [3]. The onset of the global COVID-19 pandemic in early 2020 posed additional strain on young people’s mental health, although reports on prevalence rates during this period vary. For example, increases in the prevalence and/or severity of affective and anxiety disorders [4, 5] and in self-harm and eating disorders [6] have been reported. The increased need for professional mental health care led to prolonged boarding time before inpatient admission [7]. Although the impact of the pandemic on youth mental health is complex and not fully understood, most studies are consistent in highlighting the critical need for effective treatments to meet the challenge of overburdened mental health care systems [8, 9]. Concurrently, many children and adolescents still report relatively high barriers to seeking help, with stigma and family factors being among the main perceived barriers [10, 11].

Home treatment (HT) in child and adolescent psychiatry as an alternative to inpatient treatment as usual (I-TAU) is one approach to address these challenges as it can be rapidly implemented and scaled, without the extensive infrastructure required by hospital settings [12, 13]. In contrast to I-TAU, the young patients remain in their home environment during HT, which can help address family-related barriers and reduce stigma by bringing psychiatric care to the patient, rather than the patient to the clinic [14]. Through regular and frequent visits from a multidisciplinary team – including child and adolescent psychiatrists, psychotherapists, social workers, and nursing staff – problems can be observed and addressed in the patient’s natural environment as they arise. The primary goal of HT is to empower the patient’s family, school, and social network (e.g., peers) to find sustainable solutions that support continued positive change beyond treatment discharge. Therefore, the systemic approach of HT has been suggested to increase the stability of treatment outcomes and reduce readmission rates [15, 16].

Over the past five decades, various HT approaches have been implemented as alternatives to I-TAU in child and adolescent psychiatry across Europe (e.g., Ougrin et al. [12] in the UK; Boege et al. [13] in Germany) and the USA (e.g., Henggeler et al. [17]). A recent meta-analysis provided an overview of these programs and initial empirical support for the non-inferiority of HT compared to I-TAU [18], showing comparable outcomes in psychosocial functioning and psychopathology. However, the included studies varied regarding the scope of intensity, composition of treatment teams, target populations, and whether HT fully replaced or merely supplemented hospitalization. These variations contributed to high heterogeneity in findings, limiting their generalizability and highlighting the need for further research with more standardized HT programs and evaluation strategies, especially concerning long-term outcomes [18].

In 2019, the University Hospital of Child and Adolescent Psychiatry and Psychotherapy (CAP) Bern (Switzerland) launched a pilot HT program named “AT_HOME” as an alternative to conventional I-TAU. Results of an initial pilot indicated that AT_HOME was comparably effective in reducing psychopathology during treatment [19]. The current follow-up aimed to evaluate the long-term stability of clinical outcomes and readmission rates of HT in AT_HOME compared to I-TAU. Based on the assumption that the strong systemic involvement in HT would facilitate the transfer of treatment gains into everyday life after discharge, we expected greater stability of treatment effects in the HT group, reflected in favorable follow-up outcomes and lower readmission rates.

## Methods

This original study was a monocentric, non-randomized controlled trial with two arms. Clinical data was obtained at admission and discharge within an established quality assurance process, and were previously published elsewhere [19]. Additional follow-up outcomes were assessed through clinical interviews by phone-calls between 18 and 24 months after discharge. Ethical approval was obtained from the Cantonal Ethics Committee of Bern for the retrospective use of anonymized medical records (BASEC number: REQ-2020–00546) and for the prospective follow-up study (2021-00098). The follow-up of the trial was preregistered at the “German Clinical Trials Register” (DRKS00025424, 05/27/2021).

### Population

A total of 133 children and adolescents with acute mental health disorders were admitted to the CAP Bern between May 1, 2019, and July 20, 2020, with a clinical indication for inpatient treatment, which was a prerequisite for HT in the AT_HOME program. Additional eligibility criteria included a stable housing situation within a 30-minute radius of the CAP Bern and absence of acute child welfare hazards in the patient’s home or endangerment to self or others, that required immediate protection. Of the 133 patients, 71 families (53.4%) met these criteria and could choose between HT or I-TAU; 37 families (27.8% of the original sample) opted for HT. The most common reasons for declining HT were related to acute family conflicts and families feeling overwhelmed by the demands of intensive involvement in treatment. The final sample consisted of 37 patients who received HT and 96 patients who received I-TAU. All patients were subsequently contacted and invited to participate in the current follow-up study 18 months after discharge.

### Treatment conditions

AT_HOME provided intensive psychiatric HT for up to 10 children and adolescents aged 6 to 17. Treatment typically took place in the patient’s home or relevant locations such as schools or workplaces. The program was designed to completely replace an inpatient stay for patients with a broad spectrum of psychiatric disorders (no diagnoses were excluded), which distinguishes it from less intensive outreach services, such as Multisystemic Therapy (MST). Patients received daily visits (60–120 min) six days a week, supplemented by one day with phone call only on weekends. The staffing ratio in AT_HOME was comparable to the ratio on an inpatient ward, comprising a multidisciplinary team of nurses, social pedagogues, child and adolescent psychotherapists and psychiatrists, and a school counselor (analogue to teachers in I-TAU). A 24/7 emergency hotline was available, and in cases of acute suicidal crisis, patients could be admitted to the CAP emergency unit for up to three days. Longer stays indicated dropout from AT_HOME. Treatment duration was limited to 3–4 months. The clinical details of the AT_HOME program are described in detail elsewhere [19, 20].

I-TAU was provided in one of the CAP’s five inpatient units. In Switzerland, like in other German-speaking countries, inpatient treatment is a standard option for children and adolescents with severe mental health disorders. The indication for admission is generally based on the level of psychosocial impairment or the presence of acute crises where safety concerns require close supervision, rather than based on specific disorders. While Swiss I-TAU programs often share treatment elements with outpatient care, they provide a more structured environment and intensive support for stabilization. In our study, patients generally stayed in the clinic for at least five days per week during I-TAU, with continuous staff support and daily attendance at a clinic school. Treatment duration was not fixed in I-TAU.

Both settings provided the same multimodal therapeutic approach, which included child and adolescent psychiatric case management, individual psychotherapy, and psychopharmacotherapy. Interventions were tailored to each patient’s individual needs and diagnosis-specific requirements, with consistent use of evidence-based and manualized treatment protocols. Diagnostic procedures, including physical examinations and psychopharmacology, were also applied consistently across settings.

### Outcomes & instruments

Psychopathology was assessed at admission, discharge and follow-up for all patients by clinician ratings using the Health of the Nation Outcome Scale for Children and Adolescents [HoNOSCA, 21] and for adolescents aged 12 and above by self-rating [HoNOSCA-SR, 23]. Psychosocial functioning was assessed at admission and at follow-up only using the Global Assessment of Functioning Scale [GAF, 24, 25]). A detailed description of these outcome measures and the assessment prosses are detailed in the original study [19]. Primary diagnoses were drawn from the clinic records and coded according to ICD-10 [26]. Additional outcomes at follow-up included treatment satisfaction [ZUF-8, 27] and the use of subsequent mental health services in the follow-up period collected by the “Mannheim resource module” [MRV, 22, 28]. The MRV is a tool that lists a variety of mental health services and the frequency of their use (e.g., visits, medication, hospital days) for a specific study sample and period. The original scale was modified for this study to cover all mental health-related healthcare services while excluding general health services considered less relevant to mental health. For analysis, all units were classified into three categories: inpatient treatment (including psychiatric hospitalizations, emergency treatment, and day clinics), outpatient treatment (including ambulatory care, residential homes, and other services), and medication (taken vs. not taken). Use of subsequent treatment after discharge was assessed retrospectively at follow-up.

Assessments were carried out by unblinded clinical raters at admission and discharge and via telephone by trained researchers at follow-up. Follow-up assessments were recorded and rerated by a second, blinded rater. Follow-up assessments were recorded and rerated by a second, blinded rater. Patients provided informed consent for telephone recordings with the option to request deletion of their recordings at any time. All recordings were labeled with a non-identifiable study ID and stored on Tresorit (www.tresorit.ch), a secured cloud storage solution that complies with Swiss data protection laws. Recordings were never stored on local devices. Access was restricted to four authorized team members (DG [interview lead], MK [PI], and SL [statistician]). In all analyses, the mean of the interviewer and second ratings was used. For discrepancies between raters of more than 1 category in the HoNOSCA or alternative more than 10 points on the GAF, consent was sought under supervision of an independent third senior clinician. Before consent was obtained, interrater reliability was high for HoNOSCA ratings (κ = 0.77) and very high for GAF ratings (ICC = 0.95).

### Analysis

Due to the retrospective nature of the original study design, we performed no a-priori power analyses. HoNOSCA(-SR) and GAF scores were analyzed using linear mixed models with a random intercept to group observations by subject, accounting for individual variability. The models considered the main effects of the treatment group (HT vs. I-TAU) and time points (admission, discharge, follow-up), as well as their interactions. Group by time interactions were followed by contrasts, using the Wald test, to test the hypothesized advantages of HT in achieving higher stability of the treatment effects in the HoNOSCA(-SR) and GAF at follow-up. Control variables included sex, age at study entry, and treatment duration. Post-discharge treatments were considered, including subsequent inpatient (including day clinic) and outpatient treatments, as well as medication. Interaction terms were calculated between time points and group, sex, age, and treatment duration. Due to the lack of randomization in group assignment, we implemented inverse probability weighting (IPW) [29, 30] to balance pre-treatment characteristics. Sensitivity analyses were conducted for all mixed models including only blinded second rater scores. Group differences in demographic variables were analyzed using two-tailed t-tests and Fisher’s exact tests. Missing data in the HoNOSCA were imputed using the mean of the remaining 13-*k* completed items, while only complete data records were considered for the GAF analyses. All analyses were performed using stata v17.0.

## Results

Nine patients who were admitted more than once during the study period were included only once, in the category of their first admission. Of the 34 patients eligible in the HT arm, 27 (79.4%) consented to participate in the follow-up assessment. Of the 90 patients eligible in the I-TAU arm, 48 (53.3%) participated. Detailed demographic and clinical sample characteristics are presented in Table 1. Groups did not differ regarding the distribution of sex, age at follow-up, and employment status. Similarly, treatment duration and average latency between discharge and follow-up assessment did not differ between groups. However, the distribution of primary diagnoses differed, with more affective disorders in the I-TAU group and more anxiety disorders in the HT group.

**Table 1:** Sample characteristics at follow-up

	HT (*n* = 27)	I-TAU (*n* = 48)	total (*n* = 75)	test statistics
Females, *n* (%)	13 (48%)	33 (69%)	46 (61%)	*χ*^2^(1, *N* = 75) = 3.09, *p* = 0.079
School status (in school or employed), *n* (%)	24 (88.89%)	40 (83.33%)	64 (85.33%)	*χ*^2^(1, *N* = 75) = 0.43, *p* = 0.51
Age (in years), *M* ± *SD*	15.15 ± 2.77	16.35 ± 2.87	15.92 ± 2.87	*t*(73) = 1.77, *p* = 0.081
Treatment duration (in days), *M* ± *SD*	84.59 ± 29.24	91.81 ± 58.74	89.21 ± 50.04	*t*(73) = 0.60, *p* = 0.55
Latency until follow-up (in months), *M* ± *SD*	21.33 ± 1.33	21.35 ± 2.79	21.35 ± 2.36	*t*(73) = 0.04, *p* = 0.97
Principal diagnoses at admission; *n* (%)				*χ*^2^(1, *N* = 75) = 18.34, *p* = 0.005
F1	0	1 (2.08%)	1 (1.33%)	
F2	0	3 (6.25%)	3 (4.00%)	
F3	4 (14.81%)	16 (33.33%)	20 (26.67%)	
F4	15 (55.56%)	8 (16.67%)	23 (30.67%)	
F6	0	5 (10.42%)	5 (6.67%)	
F8	2 (7.41%)	9 (18.75%)	11 (14.67%)	
F9	6 (22.22%)	6 (12.50%)	12 (16.00%)	

Note HT = Home Treatment, I-TAU = Inpatient Treatment as Usual, M = Mean, SD = Standard Deviation; principal diagnoses according to ICD-10; for a translation of the ICD-10 codes into DSM-5 diagnoses, see DSM-5 [31]

### Treatment outcome

**Fig. 1:**
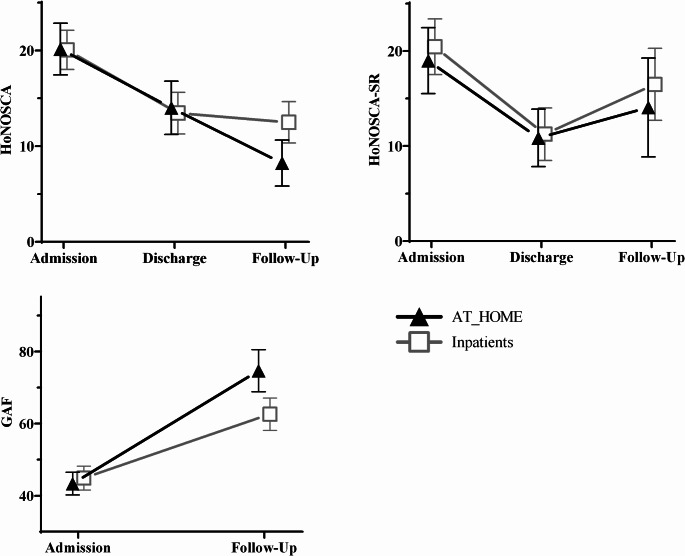
Model predictive values (Mean ± 95% confidence interval) of the trajectory of the primary outcomes in the two groups over time

Post-hoc contrasts of the group by time interactions, controlling for baseline differences and covariates, were significant for the HoNOSCA with lower scores in the HT group at follow-up (*β* = -4.25 [95%CI: -7.64 to -0.86], *SE* = 1.73, *p* = 0.014) and for the GAF with higher scores in the HT group at follow-up (*β* = 12.09 [95%CI: 4.48 to 19.70], *SE* = 3.88, *p* = 0.002), but not for the HoNOSCA-SR (*β* = -2.46, [95%CI: -9.16 to 4.30], *SE* = 3.43, *p* = 0.48). Figure 1 illustrates the model predictive values of the outcome trajectories for both groups over time. Sensitivity analyses considering the blinded second ratings did not change the results. Raw data scores of all clinical outcomes are depicted in Table S1 in the supplementary information which accompanies this article. Detailed results of the three mixed models are presented in Tables S2 – S4.

### Treatment after discharge

Utilization of subsequent care after discharge from the index treatment is presented in Table 2. Patients in the two arms did not differ in terms of the mean number of readmissions and the mean number of days in subsequent inpatient or day clinic care. There was no difference in the mean number of outpatient contacts, either. Medication use after discharge was reported by 26 (96.3%) patients in the HT arm and 39 (81.3%) patients in the I-TAU arm (Fisher’s exact test, *p* = 0.084).

**Table 2:** Subsequent care after discharge from the index treatment

	HT		I-TAU		Test statistics
	M	SD	M	SD	
Readmissions	0.81	0.79	1.00	1.09	OR = 1.23 [95%CI = 0.47 to 3.20], *p* = 0.67
Inpatient / day clinic days	75.04	99.23	55.15	92.63	*t*(73)=-0.87, *p* = 0.39
Outpatient contacts	81.85	60.71	109.92	77.91	*t*(73)=-1.61, *p* = 0.11

Note CI = Confidence Interval, HT = Home Treatment, I-TAU = Inpatient Treatment as Usual, M = Mean, OR = Odds Ratio, SD = Standard Deviation

### Treatment satisfaction

The mean satisfaction of parents in the HT arm was *M* = 16.86 ± 5.01 (*n* = 22) and in the I-TAU arm *M* = 15.92 ± 6.08 (*n* = 37), with no significant differences between groups (*t*(57) = -0.62, *p* = 0.54). The average satisfaction of patients in the HT arm was *M* = 16.17 ± 5.18 (*n* = 24) and in the I-TAU arm *M* = 13.14 ± 5.77 (*n* = 42), with significantly higher ratings in the HT arm (*t*(64) = -2.12, *p* = 0.038).

## Discussion

In this follow-up study of a trial comparing the effectiveness of HT and I-TAU for children and adolescents with psychiatric disorders, we found that patients treated with HT had significantly better outcomes in clinician-rated psychopathology and psychosocial functioning compared to those treated with I-TAU one year and a half after discharge. As reported previously [15], these results suggest that HT is particularly effective in the long-term as it facilitates the transfer of achievements during therapy after discharge due to the strong involvement of the family and the opportunity to address problems and their solutions in vivo during treatment [16, 32].

However, no significant differences were observed in self-rated psychopathology. Following initial reductions of HoNOSCA-SR scores during treatment, adolescents reported higher levels of psychopathology at follow-up compared to discharge, highlighting the challenges of transitioning from a supportive treatment environment back to everyday life. Notably, the follow-up period coincided with the early phase of the COVID-19 pandemic, which included school closures in Switzerland and other stressors repeatedly associated with adverse effects on youth mental health in national and international studies [3, 4, 33, 34]. In fact, the HoNOSCA self-ratings at follow-up in our study were comparable to scores reported in non-clinical populations during the pandemic [35], reflecting a general increase in psychological strain not observed in pre-pandemic samples [23, 36]. The discrepancy between clinician-rated and self-rated outcomes at follow-up may be partially explained in the context of the high rates of post-discharge psychiatric service use in our sample. While many adolescents may have interpreted their continued treatment as evidence of unresolved issues, clinicians might view such help-seeking behavior as a sign of resilience and resources, resulting in lower impairment ratings.

Additionally, readmission rates were high during the 18-month follow-up period in both treatment groups and no significant differences in readmission rates were found between the two treatment arms for subsequent inpatient, outpatient, and pharmacological treatment in the follow-up period. These findings are consistent with previous findings [14, 37, 38] but contrary to the expectation that higher clinical improvements in the HT arm would be reflected in a lower readmission rate. Again, the potential impact of the COVID-19 pandemic should be noted, which may have led to an overestimation of post-discharge mental health service usage in our study. Furthermore, there is evidence that adolescents with pre-existing mental health conditions may have been particularly affected during this event [39], which could have masked lower needs for subsequent psychiatric treatment after discharge in either of the treatment groups.

Acceptance of the new treatment was generally high. Parents in the HT arm reported comparable retrospective treatment satisfaction to parents in the I-TAU arm. Contrary to previous HT trials [40], patients who received HT reported higher retrospective treatment satisfaction compared to those in the I-TAU arm. This may reflect their better psychological condition at follow-up as a result of the treatment they received. Additionally, HT allowed children and adolescents (many with anxiety disorders) to avoid transfer to a psychiatric hospital, which is a stressful event for these young patients and is often perceived as stigmatizing [14, 41], which may have contributed to this higher satisfaction. Future research, including qualitative questions, could elucidate the specific advantages and disadvantages perceived by patients and thus help identify ways to improve the treatment experience.

However, the inclusion of only 27.8% of the original sample in HT highlights that this setting may not be suitable for all patients and families alike. Qualitative feedback from families who declined HT frequently cited feeling “overwhelmed as a family” or experiencing “acute family conflicts” as primary barriers, emphasizing the need for more adaptable HT models to reach broader populations. To address these barriers, one potential strategy could be the implementation of hybrid models, as proposed by Boege et al. [13] or Ougrin et al. [12]. This approach combines a short inpatient stay, providing patients and families with initial respite, with a subsequent HT phase, which focuses on family dynamics and reintegration. Another strategy could involve a higher flexibility of HT intensity. While AT_HOME is designed with a fixed treatment frequency, future programs could begin with lower intensity and frequency of visits to build therapy commitment while avoiding family burnout. Treatment frequency could increase during the main phase and taper off towards the end, addressing the need for better transitional support as demanded in previous studies [40, 42].

This study contributes to the literature on HT in child and adolescent psychiatry by using rigorous treatment protocols and standardized outcome measures (HoNOSCA[-SR] and GAF). The implementation of HT without integrated inpatient elements allows an isolated evaluation of its effects, and the non-randomized trial design offers a realistic reflection of clinical practice. In addition, with a 21-month follow-up, the study provides insights into long-term treatment effects, emphasizing a sustainable perspective on mental health interventions.

Nevertheless, our findings should be viewed in light of several limitations. First, the non-randomized, choice-based allocation may have resulted in systematic differences between groups, such as the overrepresentation of patients with anxiety disorders in the HT arm. However, a recent meta-analysis and meta-regression on the comparison between HT and I-TAU [18] revealed that HT seems to be more superior within randomized controlled trials, suggesting that our results may reflect true group differences rather than selection effects. Second, while almost 80% of eligible HT patients consented to participate, only 53% of eligible I-TAU patients did so, potentially introducing non-response bias [43, 44]. Also, a disproportionate dropout of patients with F9 disorders in the I-TAU arm reduced their representation in the follow-up sample, potentially introducing bias. While comparisons of baseline and discharge scores from the original sample to those of the reduced follow-up sample suggest minimal impact of these dropouts on outcome scores, it is unclear whether their exclusion systematically influenced follow-up outcomes. Third, the outcome measures available were limited to the assessment procedure prescribed by the Swiss ANQ initiative [45] and were rated at baseline and discharge by clinical staff who were not blinded to the treatment condition. In addition, assessment of post-discharge service usage relied on self-reported data, which is subject to recall bias. Fourth, with only 27 patients in the HT group, our sample size was too small to perform meaningful subgroup analyses to identify specific diagnoses that benefit more from HT than others. Further research with larger and balanced samples is needed to guide individualized treatment planning and replicate the current findings.

## Conclusion

Patients who received HT in the AT_HOME pilot treatment had significantly lower clinician-rated psychopathology and higher psychosocial functioning compared to patients who received I-TAU, one and a half years after discharge. These findings suggest that HT is particularly effective in the long-term and emphasize the importance of considering both short- and long-term outcomes of psychiatric treatments. Taken together, the study suggests that HT holds the potential for higher stability of treatment effects and could be one element to address the challenges faced by strained mental health systems in child and adolescent psychiatry, although open questions remain, and further research is needed.

## Electronic supplementary material

Below is the link to the electronic supplementary material.

Supplementary Material 1

## Data Availability

The data will be made available by the corresponding author upon reasonable request. The data are not publicly available due to patient privacy restrictions.
