# Supplementary material for: Comparison of the long-term outcome of home vs. inpatient treatment: 18–24 months follow-up of a non-randomized controlled trial
Source: Eur Child Adolesc Psychiatry. 2025 Mar 10;34(9):2697–705. doi: 10.1007/s00787-025-02677-x (PMC12508014; doi:10.1007/s00787-025-02677-x)
Supplement: Supplementary file 1 — Supplementary Material 1 [file 787_2025_2677_MOESM1_ESM.pdf]

Supplementary Information

Graf, Lerch, Boehnke, Reichl, Kaess. Comparison of the Long-Term Outcome of Home vs. Inpatient Treatment: 18-24 Months Follow-Up of a Non-Randomized Controlled Trial

Daniel Graf[1], Stefan Lerch[1], Ulrich Böhnke[1], Corinna Reichl[1], Michael Kaess[1,2]

[1] University Hospital of Child and Adolescent Psychiatry and Psychotherapy, University of Bern, Bern, Switzerland
[2] Department of Child and Adolescent Psychiatry, Center for Psychosocial Medicine, University of Heidelberg, Heidelberg, Germany

Correspondence
Prof. Dr. med. Michael Kaess, University Hospital of Child and Adolescent Psychiatry and Psychotherapy, University of Bern, Switzerland; email: michael.kaess@upd.ch

Table of content

Table S1. Raw scores of clinical outcomes 2
Table S2. Mixed-effects regression of the HoNOSCA outcome 3
Table S3. Mixed-effects regression of the HoNOSCA-SR outcome 4
Table S4. Mixed-effects regression of the GAF outcome 5
Table S5. CONSORT 2010 checklist of information to include when reporting a pilot trial, adapted for non-randomized trials, referring to Lancaster and Thabane (2019) 6

Table S1. Raw scores of clinical outcomes

	Home Treatment			Inpatient Treatment As Usual		
	n	*M*	*SD*	*n*	*M*	*SD*
HoNOSCA						
Admission	27	19.64	6.97	47	20.38	6.93
Discharge	27	14.13	7.26	47	13.72	6.97
Follow-Up	27	9.05	6.81	48	12.60	8.51
HoNOSCA-SR						
Admission	17	13.12	8.34	30	24.20	9.55
Discharge	17	9.29	7.33	28	12.32	9.29
Follow-Up	24	12.08	11.26	42	15.95	11.25
GAF						
Admission	27	43.04	8.15	41	45.93	12.12
Follow-Up	27	71.54	16.59	48	63.67	17.82

Note. GAF = Global Assessment of Functioning Scale, HoNOSCA(-SR) = Health of the Nation Outcome Scale for Children and Adolescents (Self-Rating), *M* = Mean, *SD* = Standard Deviation

Table S2. Mixed-effects regression of the HoNOSCA outcome

HoNOSCA	Coef.	*SE*	*z*	[95% Conf	Interval]	*p*
Time						
Postline	-7.60	1.64	-4.65	-10.81	-4.40	<0.001
Follow-Up	-14.01	2.22	-6.31	-18.37	-9.66	<0.001
Group						
AT_HOME	0.10	1.72	0.06	-3.28	3.47	0.95
Interaction						
Post#AT_HOME	0.45	1.58	0.29	-2.65	3.56	0.78
FU#AT_HOME	-4.35	2.24	-1.94	-8.73	0.03	0.052
Sex (female)	-2.02	2.03	-0.99	-6.00	1.96	0.32
Interaction						
Post#female	2.03	1.64	1.24	-1.18	5.24	0.22
FU#female	4.05	2.36	1.72	-0.58	8.67	0.086
Age	-0.18	0.28	-0.63	-0.74	0.38	0.53
Interaction						
Post#age	0.10	0.28	0.34	-0.46	0.65	0.73
FU#age	0.05	0.36	0.14	-0.66	0.76	0.89
Treatment Duration	0.02	0.02	0.97	-0.02	0.05	0.33
Interaction						
Post#Treatment Duration	-0.06	0.02	-3.49	-0.09	-0.02	<0.001
FU#Treatment Duration	-0.08	0.02	-3.81	-0.13	-0.04	<0.001
Inpatient days in FU period	0.01	0.01	0.82	-0.01	0.03	0.41
Outpatient contacts in FU period	0.02	0.01	1.22	-0.01	0.04	0.22
Medication in FU period (yes/no)	2.80	1.64	1.71	-0.41	6.01	0.088
Constant	21.18	1.85	11.42	17.55	24.82	<0.001

Note. HoNOSCA = Health of the Nation Outcome Scale for Children and Adolescents, *SE* = Standard Error

Table S3. Mixed-effects regression of the HoNOSCA-SR outcome

HoNOSCA-SR	Coef.	*SE*	*z*	[95% Conf	Interval]	*p*
Time						
Postline	-6.19	2.58	-2.40	-11.25	-1.14	0.016
Follow-Up	-7.57	4.03	-1.88	-15.48	0.33	0.060
Group						
AT_HOME	-1.45	2.32	-0.63	-5.99	3.09	0.53
Interaction						
Post#AT_HOME	1.06	2.66	0.40	-4.15	6.26	0.69
FU#AT_HOME	-0.98	4.01	-0.24	-8.83	6.88	0.81
Sex (female)	6.05	2.32	2.61	1.50	10.60	0.009
Interaction						
Post#female	-0.94	2.82	-0.33	-6.47	4.60	0.74
FU#female	7.02	3.20	2.20	0.75	13.29	0.028
Age	2.75	0.74	3.74	1.31	4.19	<0.001
Interaction						
Post#age	-1.83	0.67	-2.75	-3.14	-0.53	0.006
FU#age	-2.37	0.91	-2.60	-4.15	-0.58	0.009
Treatment Duration	-0.01	0.03	-0.47	-0.07	0.05	0.64
Interaction						
Post#Treatment Duration	-0.06	0.03	-1.91	-0.11	0.01	0.056
FU#Treatment Duration	-0.10	0.05	-1.83	-0.21	0.01	0.067
Inpatient days in FU period	0.02	0.01	1.16	-0.01	0.04	0.25
Outpatient contacts in FU period	-0.02	0.03	-0.97	-0.07	0.03	0.33
Medication in FU period (yes/no)	3.91	4.37	0.90	-4.65	12.47	0.37
Constant	13.31	2.12	6.27	9.15	17.48	<0.001

Note. HoNOSCA-SR = Health of the Nation Outcome Scale for Children and Adolescents – Self-Rating, *SE* = Standard Error

Table S4. Mixed-effects regression of the GAF outcome

GAF	Coef.	SE	*z*	[95% Conf	Interval]	*p*
Time						
Follow-Up	38.25	4.85	7.89	28.74	47.75	<0.001
Group						
AT_HOME	-1.51	2.31	-0.66	-6.03	3.01	0.51
Interaction						
FU#AT_HOME	13.60	4.44	3.06	4.89	22.31	0.002
Sex						
Female	-2.50	2.73	-0.92	-7.84	2.84	0.36
Interaction						
FU#Female	-3.60	4.78	-0.75	-12.97	5.78	0.45
Age	-.89	0.39	-2.31	-1.65	-0.14	0.021
Interaction						
FU#Age	1.95	0.78	2.50	0.42	3.47	0.012
Treatment Duration	0.01	0.03	0.38	-0.04	0.06	0.70
Interaction						
FU#Treatment Duration	0.11	0.05	2.34	0.02	0.20	0.019
Inpatient days in FU period	-0.04	0.02	-1.57	-0.08	0.01	0.12
Outpatient contacts in FU period	-0.03	0.03	-1.11	-0.09	0.02	0.27
Medication in FU period (yes/no)	-15.11	3.77	-4.01	-22.50	-7.72	<0.001
Constant	46.20	2.62	17.64	41.07	51.33	<0.001

Note. GAF = Global Assessment of Functioning Scale, *SE* = Standard Error

Table S5. CONSORT 2010 checklist of information to include when reporting a pilot trial, adapted for non-randomized trials, referring to Lancaster and Thabane (2019)

**Section/Topic**	**Item No**	**Checklist item**	**Reported on page No**
**Title and abstract**			
	1a	Identification as a non-randomised trial in the title	1
	1b	Structured summary of trial design, methods, results, and conclusions	2
**Introduction**			
Background and objectives	2a	Scientific background and explanation of rationale for future definitive trial, and reasons for non-randomised trial	3-4
	2b	Specific objectives or research questions for trial	4
**Methods**			
Trial design	3	Description of trial design (such as parallel, factorial)	4
Participants	4a	Eligibility criteria for participants	4-5
	4b	Settings and locations where the data were collected	4-5
	4c	How participants were identified and consented	4-5
Interventions	5	The interventions for each group with sufficient details to allow replication, including how and when they were actually administered	5
Outcomes	6a	Completely defined prespecified assessments or measurements to address each trial objective specified in 2b, including how and when they were assessed	5-6
Sample size	7a	Rationale for numbers in the trial	6
	7b	When applicable, explanation of any interim analyses and stopping guidelines	Not applicable
Randomisation:			
Sequence generation	8a	Method used to generate the random allocation sequence	No randomization
	8b	Type of randomisation(s); details of any restriction (such as blocking and block size)	
Allocation concealment mechanism	9	Mechanism used to implement the random allocation sequence (such as sequentially numbered containers), describing any steps taken to conceal the sequence until interventions were assigned	
Implementation	10	Who generated the random allocation sequence, who enrolled participants, and who assigned participants to interventions	
Blinding	11a	If done, who was blinded after assignment to interventions (for example, participants, care providers, those assessing outcomes) and how	

	11b	If relevant, description of the similarity of interventions	
Statistical methods	12	Methods used to address each objective whether qualitative or quantitative	6-7
**Results**			
Participant flow (a diagram is strongly recommended)	13a	For each group, the numbers of participants who were approached and/or assessed for eligibility, randomly assigned, received intended treatment, and were assessed for each objective	No randomization
	13b	For each group, losses and exclusions after randomisation, together with reasons	
Recruitment	14a	Dates defining the periods of recruitment and follow-up	7
	14b	Why the trial ended or was stopped	Not applicable
Baseline data	15	A table showing baseline demographic and clinical characteristics for each group	7
Numbers analysed	16	For each objective, number of participants (denominator) included in each analysis. If relevant, these numbers should be by randomised group	7
Outcomes and estimation	17	For each objective, results including expressions of uncertainty (such as 95% confidence interval) for any estimates. If relevant, these results should be by randomised group	7-8
Ancillary analyses	18	Results of any other analyses performed that could be used to inform the future definitive trial	Not applicable
Harms	19	All important harms or unintended effects in each group (for specific guidance see CONSORT for harms)	No adverse events
**Discussion**			
Limitations	20	Limitations, addressing sources of potential bias and remaining uncertainty about feasibility	10-11
Generalisability	21	Generalisability (applicability) of methods and findings to future definitive trial and other studies	10-11
Interpretation	22	Interpretation consistent with objectives and findings, balancing potential benefits and harms, and considering other relevant evidence	9-10
	22a	Implications for progression to future definitive trial, including any proposed amendments	11
**Other information**			
Registration	23	Registration number for trial and name of trial registry	4
Protocol	24	Where the trial protocol can be accessed, if available	No protocol
Funding	25	Sources of funding and other support (such as supply of drugs), role of funders	12
	26	Ethical approval or approval by research review committee, confirmed with reference number	4

Lancaster, G. A., & Thabane, L. (2019). Guidelines for reporting non-randomised pilot and feasibility studies. *Pilot Feasibility Stud, 5*, 114. doi: https://doi.org/10.1186/s40814-019-0499-1
